# Intraoperative radiation therapy for advanced cervical metastasis: a single institution experience

**DOI:** 10.1186/1748-717X-6-72

**Published:** 2011-06-15

**Authors:** Youssef H Zeidan, Alex Yeh, Daniel Weed, Colin Terry, Stephen Freeman, Edward Krowiak, Robert Borrowdale, Tod Huntley

**Affiliations:** 1 Department of Radiation Oncology, Stanford University, Stanford, CA, USA; 2 Department of Radiation Oncology, Methodist Hospital, Indianapolis, IN, USA; 3 Center for Ear Nose Throat & Allergy, Indianapolis, IN, USA; 4 Methodist Research Institute, Methodist Hospital, Indianapolis, IN, USA

**Keywords:** intraoperative radiotherapy, IORT, cervical metastasis

## Abstract

**Background:**

The purpose of this study is to review our experience with the use of IORT for patients with advanced cervical metastasis.

**Methods:**

Between August 1982 and July 2007, 231 patients underwent neck dissections as part of initial therapy or as salvage treatment for advanced cervical node metastases resulting from head and neck malignancies. IORT was administered as a single fraction to a dose of 15 Gy or 20 Gy in most pts. The majority was treated with 5 MeV electrons (112 pts, 50.5%).

**Results:**

1, 3, and 5 years overall survival (OS) after surgery + IORT was 58%, 34%, and 26%, respectively. Recurrence-free survival (RFS) at 1, 3, and 5 years was 66%, 55%, and 49%, respectively. Disease recurrence was documented in 83 (42.8%) pts. The majority of recurrences were regional (38 pts), as compared to local recurrence in 20 pts and distant failures in 25 pts. There were no perioperative fatalities.

**Conclusions:**

IORT results in effective local disease control at acceptable levels of toxicity. Our results support the initiation of a phase III trial comparing outcomes for patients with cervical metastasis treated with or without IORT.

## Background

The management of advanced or recurrent cervical node metastases poses a challenge for surgeons and radiation oncologists. In general, primary tumor sites which are drained by a dense lymphatic supply, such as the nasopharynx and hypopharynx, are more prone to cervical spread compared to tissues with more limited lymphatics, such as the paranasal sinuses, middle ear, and true vocal folds [1]. In addition to the primary site's lymphatic supply, the risk of cervical node metastasis rises directly with the size of the primary tumor and inversely with its histologic differentiation [2].

Complete resection of cervical node metastases is not always feasible due to tumor proximity to vital structures such as the carotid artery or to fixation to deep tissues such as the prevertebral fascia. In addition, prior surgery and radiation therapy can induce tissue fibrosis and alter the anatomy sufficiently to result in recognized or unknown gross or microscopic residual neck disease.

Intraoperative radiation therapy has been available to select head and neck cancer patients presenting to our group since the 1980s [3,4]. IORT has been offered to those patients who have metastatic nodal disease recurrent or persistent after prior surgery and/or radiation treatment or who have nodal disease at initial presentation which in the judgment of the surgeon has a significant chance of having gross or residual microscopic cancer persistent at the conclusion of the surgery. The IORT is delivered to the tumor bed following surgical extirpation. The method of radiation at the time of surgery allows for effective shielding and retraction of critical structures such as the cervicofacial skin, laryngopharynx, and mandible, while allowing for maximal exposure of the tumor bed to the radiation beam.

IORT offers several radiobiologic advantages including decreased tumor repopulation and improved targeting of hypoxic portions of residual tumor [5-7]. IORT is especially helpful in neck disease as a boost for adjuvant EBRT. Cons include the theoretical induction of fibrosis of late responding tissues, the need for additional manpower in the operating room, and the extension of the operative time by approximately 45 minutes.

The current study updates our previously reported experience with management of advanced cervical metastasis using IORT and neck dissection [8,9]. This analysis includes evaluation of clinical outcomes of integrating IORT in treatment of advanced cervical metastasis with analysis of potential prognostic factors.

## Materials and methods

### Study population

Between August 1982 and July 2007, 231 patients were treated with surgery and IORT for advanced cervical node metastases from head and neck cancers as part of initial treatment or for recurrent disease. This was a very small subset of the general population undergoing neck surgery as part of the treatment of head and neck malignancies. Patient demographics are summarized in table 1. Sixty-one (26.4%) were females and 170 (73.6%) were males. The median age of the patient population at the time of primary or salvage surgery with IORT was 63.5 years (range 32.9 to 90.3 yrs). All of these cases presented with extensive neck disease that had high chance for lymphovascular or perineural spread, extracapsular extension, or extension to surrounding the deep neck musculature, prevertebral fascia, carotid artery, or other vital structures that in the opinion of the treating surgeon might preclude definitive surgical removal with negative margins and no residual microscopic disease. Simple invasion of resectable muscles such as the sternocleidomastoid muscle, cranial nerves XI or XII, the internal jugular vein, etc. were not criteria for IORT treatment by themselves; such structures were resected using standard surgical principles and IORT would not necessarily have been offered.

**Table 1:** Patient Characteristics

Characteristic	N (%)
Gender	
Male	170 (73.6%)
Female	61 (26.4%)
Prior Chemo (yes)	99 (50.5%)
Prior RT (yes)	175 (81.4%)
Surgery Type	
Primary	26 (11.6%)
Salvage	198 (88.4%)

General indications for treatment included: 1) tumor that could not be dissected with obviously clean margins from vital nerves, muscles, the carotid artery, or bony structures 2) disease which was thought to be more aggressive than usual, 3) large or bulky disease or N3 nodes, 4) suspected close or positive margins or cases with suspected residual microscopic disease and 5) prior full course external beam radiotherapy. If the neck disease could be removed without significant risk of residual microscopic or gross disease, IORT was not considered. The study was performed in accordance with the Declaration of Helsinki and approved as a retrospective review by the Institutional Review Board at Methodist Hospital of Indiana. Characteristics of the study population are summarized in Table 1.

### Treatment Methods

All patients were treated by members of a single surgical practice and a single radiation oncology group. Computed tomography (CT) scanning of the head and neck was performed on all patients and the images were reviewed preoperatively by the treating physicians. The majority of the patients had previously undergone treatment to the neck with either surgery, radiation, or both. Surgery with IORT was performed for salvage in 198 patients and 26 patients had not been treated previously. One patient received 10 Gy, two received 12 Gy, 1 received 13 Gy, 83 received 15 Gy, 1 received 17 Gy, 1 received 17.5 Gy, 3 received 18 Gy, 132 received 20 Gy, and 5 received 25 Gy, all prescribed to the maximum isodose line. Although the ideal IORT dose is yet to be determined, prior experience indicates higher incidence of complications with IORT doses above 20 Gy in HNC pts (24). Considerations for dose selection in our study included tumor size, location and prior treatment.

The neck dissections were performed via standard surgical principles. After the resection was completed, the radiation oncologist entered the operating room to assist with the IORT portion of the procedure.

There was no single dose, cone size, or electron energy used for all treatments. Median treatment cone size was 6.4 cm, ranging from 3 cm to 10.2 cm. As for beam energy 65 (29.8%),112 (50.5%), and 45 patients (20.3%) were prescribed 4, 5, and 6 MeV electrons respectively, dosed to D_max_. There were 88 patients (39.1%) who received 15 Gy or less and 142 (60.9%) patients who received more than 15 Gy.

Postoperative EBRT was prescribed to 50 patients at the discretion of the attending radiation oncologist. Median dose was 45 Gy (range, 20-66 Gy). Overall, 99 patients received chemotherapy (adjuvant, palliative, neoadjuvant, etc.). Follow-up consisted of clinical examinations with radiographic follow-up as clinically indicated.

### Statistical analyses

The endpoints analyzed were overall survival (OS), recurrence-free survival (RFS), and local control (LC). All events were measured from the date of primary or salvage surgery with IORT. Local recurrence was defined as evidence of recurrent disease in the IORT field. Failures outside the IORT field but within or adjacent to the surgical bed were considered regional. One-, 3-, and 5-year estimates of OS and RFS were derived using the Kaplan-Meier method, with comparisons among groups performed with 2-sided log-rank tests. A Cox proportional hazards model was used to identify characteristics predictive of survival and disease recurrence. All tests were two-tailed comparisons, and the acceptable probability of a type I error was set as less than 0.05 for statistical significance.

## Results

### Tumor characteristics

Tumor characteristics are summarized in Table 2. Median neck tumor size was 4.3 cm. The majority of the tumors (90.9%) were squamous cell carcinoma (SCC) arising in the upper aerodigestive tract. Nearly half of the neck lesions were on right side (n = 114, 49.4%), 39.4% were left-sided (n = 91) 9.1% (n = 21) were bilateral and 2.2% (n = 5) presented in the anterior midline.

**Table 2:** Tumor Characteristics

Characteristic	N (%)
**Tumor margins**	
Close Margin	8 (3.4%)
Negative Margin	129 (55.6%)
Positive Margin	41 (17.7%)
Margins Unknown	54 (23%)
**Histology**	
Squamous Cell Carcinoma	210 (90.9%)
Other	21 (9.1%)
**Side of Neck for IORT**	
Anterior	5 (2.2%)
Right	114 (49.4%)
Left	91 (39.4%)
Bilateral	21 (9.1%)
Perineural spread	30 (16.9%)
Lymphovascular involvement	29 (16.3%)
Extracapsular extension	22 (12.3%)
Vascular Invasion	27 (15.1%)
Dermal Invasion	37 (20.7%)
Carotid Involvement	60 (32.6%)

Surgical margins of the neck disease were grossly or microscopically positive per frozen section in 41 pts (23.0%), close (generally defined as tumor within 1 mm to the margin) in 8 pts (4.5%) and histologically negative per frozen section in 129 pts (72.5%). Lymphovascular invasion (LVI) and perineural invasion were observed in 29 pts (16.3%) and 30 pts (16.9%), respectively. Extracapsular extension (ECE) and dermal invasion were noted in 22 (12.3%) and 37 pts (20.7%) respectively. Carotid artery involvement was noted in 60 pts (32.6%).

### Overall Survival

**Figure 1:**
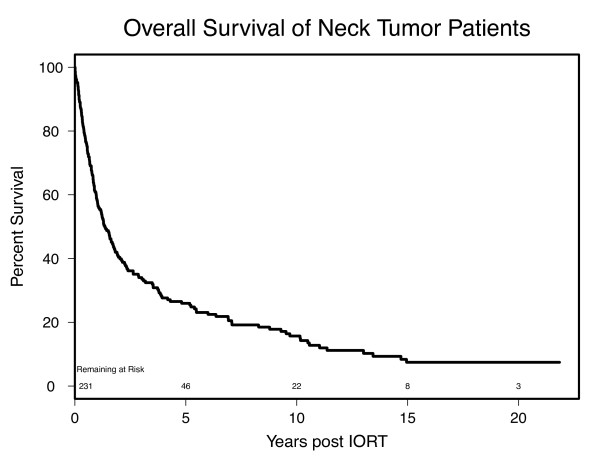
**Kaplan-Meier estimates showing overall survival rates for patients undergoing cervical IORT**.

With a median follow up of 1.03 yrs (range 0.01 to 21.85 yrs), 53 patients were known to be alive at the time of this analysis. The 1-, 3- and 5- year survival rates (Figure 1) were 58%, 34%, and 26%, respectively. Table 3 shows that patients with carotid involvement had significantly worse survival with a median survival of 1 year compared to 2.2 years for patients with uninvolved carotids (p = 0.01). Pathological features such as perineural and dermal invasion were also predictive of decreased survival (p < 0.001 and p = 0.035 respectively). Survival outcomes were not significantly altered by margin status, dose delivered (< 15 Gy or > 15 Gy), beam energy (4, 5 or 6 MeV), prior chemotherapy, or prior RT treatment.

**Table 3:** Statistical correlation of disease characteristics with survival outcomes

Characteristic (%)	Median OS (y)	*p*	Median RFS (y)	*p*
**IORT dose**		0.863		0.029
≤ 1500 cGy (39.1%)	1.2		1.5	
> 1500 cGy (60.9%)	1.5		NE	
**Energy**		0.064		0.006
4 MeV (29%)	1.0		NE	
5 MeV (51%)	1.6		NE	
6+ MeV (20%)	1.0		0.8	
**ECE**		0.569		0.310
Yes (12%)	1.4		0.9	
No (88%)	1.7		3.9	
**LVI/AVI**		0.071		0.064
Yes (16%)	0.8		0.7	
No (84%)	1.7		3.9	
**PNI**		< 0.001		0.387
Yes (17%)	0.6		1.1	
No (83%)	1.9		3.9	
**Dermal Invasion**		0.035		0.911
Yes (21%)	0.9		NE	
No (79%)	1.9		3.1	
**Carotid Involvement**		0.010		0.199
Yes (33%)	1.0		1.1	
No (67%)	2.2		NE	
**Vasc. Complications**		0.823		0.894
Yes (11%)	1.2		1.1	
No (89%)	1.2		1.5	
**Prior RT**		0.263		0.246
Yes (81%)	1.4		3.2	
No (19%)	2.2		NE	
**Previous Chemo**		0.419		0.045
Yes (51%)	1.6		1.2	
No (49%)	0.9		10.4	
**Post Surgery RT**		0.457		0.127
No (76%)	1.5		10.4	
Yes (24%)	1.6		1.2	

Survival times estimated using Kaplan-Meier method and tested between groups using the long-rank test.

NE - not estimable

### Local control, recurrence, and recurrence-free survival

**Figure 2:**
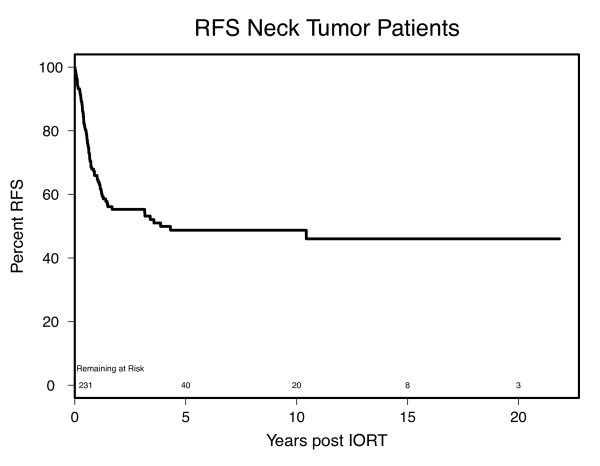
**Kaplan-Meier estimates showing recurrence free survival rates for patients undergoing cervical IORT**.

Recurrence-free survival (RFS) is shown in Figure 2. RFS at 1, 3, and 5 years was 66%, 55%, and 49%, respectively. A significantly shorter time to recurrence was noted in patients who had prior chemotherapy as compared to chemotherapy naïve patients (1.2 vs. 10.4 years p = 0.045), which was thought to be reflective of the extent of disease at initial presentation rather than due to the chemotherapy itself, though this is unproven. Patients treated with doses above 15 Gy had significantly improved overall RFS (p = 0.029), but as noted above, no improvement in OS was noted with dose level. None of the other studied factors including margin status, prior RT, postoperative RT, carotid involvement, dermal invasion, perineural or vascular invasion significantly impacted RFS.

Thirty eight patients (16%) experienced regional recurrence and twenty patients (9%) had local recurrence. Distant metastases were later detected in twenty five patients (11%). Fifty seven patients (25%) failed within the surgical field. Of those, only twenty patients (9%) failed within the IORT field.

### Complications

There were no perioperative fatalities. Complications data was available on 203 pts. Postoperative complications occurred in 54 pts resulting in 80 complication events. As shown in Table 4, there were 23 vascular complications. Among those, there were 10 strokes and 6 hematomas. Other vascular complications included TIA, carotid blow out, and cardiac ischemic events. There were 20 pharyngocutaneous fistulas developed within the first few weeks of surgery and 20 postoperative wound dehiscence events. Sensory neuropathy developed in 7 cases, 8 pts developed radiation osteonecrosis, and in 2 pts there was partial necrosis of a reconstructive flap. Mean IORT dose in pts with no complications was 18.18 Gy vs. 17.77 Gy in pts with at least one complication. We found no significant correlation between IORT dose delivered and complication risk (p = 0.361).

**Table 4:** Complications

Complications (203 pts with available data)	N (%)
Vascular Complications	23
Radiation osteonecrosis	8
Fistulas	20
Flap Necrosis	2
Wound dehiscence	20
Neuropathy	7
**Total events**	80
Pts with > 1 complication	54 (27%)
Pts with no complications	149 (73%)

## Discussion

Advanced cervical metastasis presents significant challenges to both the head and neck surgeon and the radiation oncologist. Despite advances in surgical and radiation techniques, survival rates in for patients with advanced cervical metastasis remains low.

From a radiobiology standpoint, IORT allows delivery of a high dose of electron beam energy directly to the region of greatest risk. A single IORT dose is biologically equivalent to two to three times the same dose delivered via EBRT [5]. In addition, the proximity of IORT to the time of resection may be advantageous; Ang et al. reported improved survival and locoregional control when patients with advanced head and neck cancer received radiation within 11 weeks postoperatively [10].

The use of IORT for head and neck cancer has been limited to a handful of institutions. Recently, Chen et al reported the UCSF experience with 137 pts treated for recurrent head and neck cancer. Their 3-year in-field control rate and overall survival rate were 67% and 36%, respectively [11]. In another study, Pinheiro and colleagues reported their results for 44 patients treated at Mayo clinic. Overall survival and disease free survival were 32% and 21% for pts with SCC and 50% and 40% for pts treated for other histologies [12]. Lastly, a retrospective study of 38 patients treated at Ohio State with IORT for recurrent head and neck cancer found that neck IORT was accompanied by improved overall survival [13]. In our retrospective series, the OS and RFS were 26% and 49% respectively at 5 years. While our numbers compare favorably to the literature, one has to keep in mind the probable inherent heterogeneity of the different study populations. As previously noted and as summarized in Table 3 our inclusion criteria for this study was advanced neck disease that in many institutions would have been deemed poorly resectable or even unresectable, with nearly a third of the patients presenting with frank carotid involvement, 20.7% of the patients with dermal/skin involvement, and nearly half with extracapsular spread, Lymphovascular involvement, and/or perineural spread. The results from this study must be looked at with this in mind. The majority of these patients were at high risk for the development of distant metastatic disease and for failure at the primary upper aerodigestive site, as well as in the neck.

One major limitation of this study is its retrospective nature, which by definition limits data availability and analysis. Furthermore, it is difficult to sort out the benefit attributable to IORT in this population because some patients received a variety of adjuvant and neoadjuvant chemotherapy and radiation therapy courses in addition to neck dissection with IORT.

In a prior study we identified gross residual disease as a predictor of poor patient outcome after IORT [9]. In the current report, patients with carotid involvement had a dismal median OS of 1 year. This reflects the previously reported high complication rates of 50% in these patients [8]. This subset of patients is at high risk for post-treatment cerebrovascular events and neurologic sequelae.

Several studies have confirmed better disease control when IORT is used in conjunction with EBRT. Nag et al reported 79% local control in pts who received additional EBRT vs. 50% for those who had IORT alone [14,15]. In the current series there were 50 pts (24%) who received post treatment RT. However, there was no statistically significant difference noted for OS and RFS for those pts. Perhaps this can be explained by the relatively high number of pts who had prior RT (175 pts) in this group.

Postoperative complications occurred in 54 pts (27%.) The majority of these complications were not thought to be due to the IORT itself, however, but were thought instead to be reflective of the scope of the surgery in general for these patients with advanced disease, many with cancer recurrent or persistent after prior surgery, RT, and chemotherapy. The majority of the patients, (n = 175) had undergone previous RT and 50 were given postoperative RT, so some patients were re-irradiated. In addition, 99 patients had previously been treated with chemotherapy. The surgical complication rate in such a population is high in general [8,16], regardless of whether IORT is offered or not. This population is at high risk for wound dehiscence and postoperative pharyngocutaneous fistula formation, and the 20 cases we experienced in each category were not thought to be a result of the IORT. In each case, the skin that dehisced had been shielded with lead and was not exposed to the radiotherapy beam. Likewise, the pharyngeal mucosa at the postoperative fistula sites had been appropriately shielded with lead. Similarly, the partial flap necrosis in 2 of the pts was in non-irradiated tissue which should not have been affected by the IORT.

Bearing in mind the number of patients with unfavorable features included in the study (Table 2), our complication rate of only 27% is acceptable. Reported experiences with IORT in HNC pts has major complications ranging from 6.5% to 28.4% (6, 7, 24-26). Such complications are likely multifactorial in etiology including tumor invasion of critical structures and prior treatments in addition to the treatment delivered. Although the ideal IORT dose is yet to be determined, prior experience indicates higher incidence of complications with IORT doses above 20 Gy in HNC pts (24). In addition to dose other factors that need to be considered inorder to minimize complications include: cone size, proper shielding and patient comorbidities. The current series is the largest reported to date which addresses the role of IORT in advanced cervical disease. The reported 5 year OS of 26% and RFS of 49% compare favorably to historical controls. Future efforts should be directed to improve disease control by decreasing regional and distant failures. The current study also identifies clinical factors that correlate with better outcomes. Such prognostic factors are important for refining patient selection for IORT in the future. Our retrospective analysis supports incorporation of IORT into future randomized phase III clinical trials to improve outcomes in patients with advanced cervical metastasis

## Competing interests

The authors declare that they have no competing interests.

## Authors' contributions

YHZ analyzed the data and wrote the manuscript. He is the corresponding author. AY reviewed the manuscript and the data analysis. CT participated in statistical analysis. DW, SF, EK and RB contributed to discussion and data analysis. TH participated in data analysis and manuscript writing. All the authors read and approved the final manuscript.
